# Fibroblast Growth Factor Receptor Signaling in Skin Cancers

**DOI:** 10.3390/cells8060540

**Published:** 2019-06-04

**Authors:** Malgorzata Czyz

**Affiliations:** Department of Molecular Biology of Cancer, Medical University of Lodz, 6/8 Mazowiecka Street, 92-215 Lodz, Poland; malgorzata.czyz@umed.lodz.pl; Tel.: +48-42-272-57-02

**Keywords:** FGF, fibroblast growth factor, FGFR, autocrine signaling, skin, melanoma, squamous and basal cell carcinoma, seborrheic keratosis, targeted therapy, resistance

## Abstract

Fibroblast growth factor (FGF)/Fibroblast growth factor receptor (FGFR) signaling regulates various cellular processes during the embryonic development and in the adult organism. In the skin, fibroblasts and keratinocytes control proliferation and survival of melanocytes in a paracrine manner via several signaling molecules, including FGFs. FGF/FGFR signaling contributes to the skin surface expansion in childhood or during wound healing, and skin protection from UV light damage. Aberrant FGF/FGFR signaling has been implicated in many disorders, including cancer. In melanoma cells, the FGFR expression is low, probably because of the strong endogenous mutation-driven constitutive activation of the downstream mitogen-activated protein kinase-extracellular signal-regulated kinase (MAPK-ERK) signaling pathway. FGFR1 is exceptional as it is expressed in the majority of melanomas at a high level. Melanoma cells that acquired the capacity to synthesize FGFs can influence the neighboring cells in the tumor niche, such as endothelial cells, fibroblasts, or other melanoma cells. In this way, FGF/FGFR signaling contributes to intratumoral angiogenesis, melanoma cell survival, and development of resistance to therapeutics. Therefore, inhibitors of aberrant FGF/FGFR signaling are considered as drugs in combination treatment. The ongoing LOGIC-2 phase II clinical trial aims to find out whether targeting the FGF/FGFR signaling pathway with BGJ398 may be a good therapeutic strategy in melanoma patients who develop resistance to v-Raf murine sarcoma viral oncogene homolog B (BRAF)/MEK inhibitors.

## 1. Introduction

**Figure 1:**
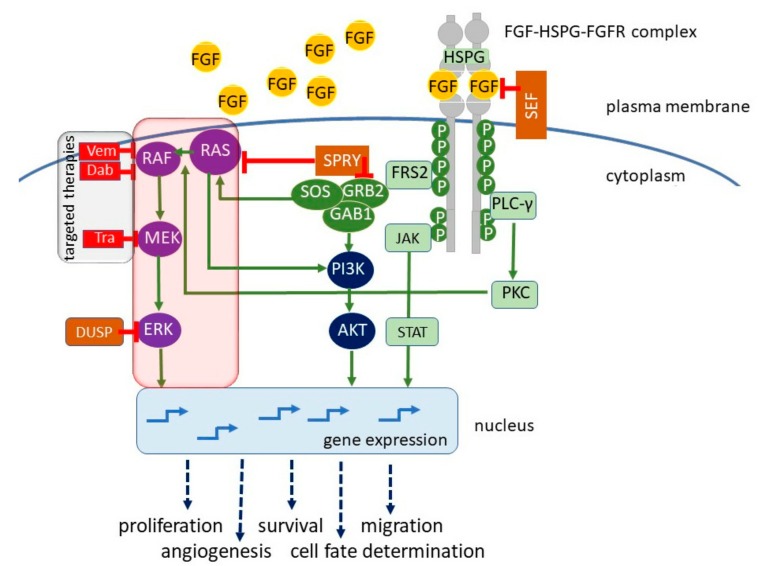
Fibroblast growth factor receptors (FGFRs) are highly conserved transmembrane receptors consisting of three extracellular immunoglobulin-like (Ig-like) domains, a transmembrane helical region, and a cytoplasmic region with kinase activity. The fibroblast growth factor (FGF) ligand and its cofactor heparin sulfate proteoglycan (HSPG) bind to FGFR monomers, leading to dimerization and tyrosine cross-autophosphorylation of the cytoplasmic domain. This induces various signaling pathways, resulting in cellular proliferation, survival, migration, angiogenesis, and cell fate determination in embryogenesis and in response to microenvironmental signals, including therapeutics. FGF/FGFR signaling can be stimulated in a paracrine manner, mainly in physiological settings, or in an autocrine manner as demonstrated in various cancers. In melanoma, FGF/FGFR signaling is largely suppressed by mutation-driven enhanced activity of the RAS (Rat sarcoma oncogene)/BRAF (v-Raf murine sarcoma viral oncogene homolog B)/MEK (mitogen-activated protein kinase)/ERK (extracellular signal-regulated kinase) pathway (red framed). Melanoma cells that acquire the ability to secrete FGFs and stimulate FGFR in a paracrine or autocrine manner can contribute to angiogenesis and cell-fate decisions involving transitions between different phenotypes, including phenotypes resistant to targeted therapies (grey framed). Dab, dabrafenib; DUSP, dual-specificity phosphatase; FRS2, FGFR substrate 2; GAB1, GRB2-associated binding protein 1; GRB2, growth factor receptor protein 2; JAK, Janus kinase; PKC, protein kinase C; PLC-γ, phospholipase C gamma; SOS, son of sevenless; SEF, similar expression to FGF; SPRY, Sprouty; STAT, signal transducer and activator of transcription; Tra, trametinib; Vem, vemurafenib.

Receptor tyrosine kinases (RTKs) are cell membrane proteins comprising about 20 families with nearly 60 members. The Fibroblast Growth Factor Receptor (FGFR) family of human RTKs consists of four highly conserved transmembrane receptors (FGFR1–4) and one FGF receptor without an intracellular domain (FGFR5). FGFRs are mainly localized at the cell surface; however, they are also present inside of the cells, in the nucleus and mitochondria [1]. The common structure of cell membrane-localized FGFRs consists of a large ligand-binding extracellular region with three immunoglobulin-like (Ig-like) domains, a transmembrane helical region, and a cytoplasmic region with a catalytically active tyrosine kinase domain. The alternative splicing of the third Ig-like domain results in a variety of FGFR isoforms with different ligand specificities [2,3]. FGFR activity is modulated in diverse ways, including posttranslational modifications and formation of complexes with selected ligands and other cell membrane proteins [4]. Over 20 distinct fibroblast growth factors (FGFs) have been identified as the ligands of FGFRs. Binding of FGFs to FGFRs is assisted by cofactors, heparin sulfate proteoglycans (HSPGs) in paracrine FGF signaling, or Klotho coreceptor in endocrine signaling [5]. It triggers the dimerization of receptor monomers in the membrane and cross-autophosphorylation of tyrosine residues in the cytoplasmic kinase domain, which is followed by binding of various downstream effector molecules, including phospholipase C gamma (PLCγ), fibroblast growth factor receptor substrate 2 (FRS2), son of sevenless (SOS), and growth factor receptor-bound 2 (GRB2). FRS2 is an adaptor/scaffold protein, which acts downstream of a limited number of RTKs, including FGFR [6]. *FRS2* was annotated as amplified in skin-derived tumors in the Cancer Genome Project dataset [7,8] and emerged as a potential therapeutic target in melanoma [9]. Binding of adaptor proteins induces the activation of several signaling pathways, such as protein kinase C (PKC), mitogen-activated protein kinase-extracellular signal-regulated kinase (MAPK-ERK), phosphoinositide 3-kinase/protein kinase B (PI3K/AKT), and signal transducer and activator of transcription 3/5 (STAT3/5) signaling pathway (Figure 1).

FGFR triggered signaling pathways play crucial roles in morphogenesis during embryonic development [10]. Signaling from FGFR is also important for controlling the nervous system, angiogenesis, metabolism, endocrine function, wound healing, and tissue repair in the adult organism. FGF signaling regulates expression of genes by modulating microRNA abundance [11,12]. Besides being involved in normal development, abnormal activities of FGRFs has been documented in hereditary diseases and a wide range of cancers [13,14,15,16,17]. In the recently performed large-scale high-throughput study, the dysregulation of FGFRs was found in 7% of cancers [18].

Melanoma belongs to a group of highly lethal cancers. Several signaling pathways are constitutively activated in melanoma [19]. Recently developed technologies, including next-generation sequencing (NGS), led to a new genetic-based classification of melanoma [20,21]. The activity of the MAPK signaling pathway, comprising the cascade of RAS (Rat sarcoma oncogene)/RAF (v-Raf murine sarcoma viral oncogene homolog B)/MEK (mitogen-activated protein kinase kinase)/ERK, is very frequently altered in melanoma by somatic mutations [22]. About 50% of melanoma patients harbor activating mutations in *BRAF* with BRAF^V600E^ as the main protein product, whereas *NRAS* is mutated in about 15–20% of cases [23,24]. The constitutive activity of the MAPK signaling pathway results in elevated proliferation rate and enhanced survival potential of melanomas. Therefore, in addition to the development of immunotherapies, the main effort is focused on targeted therapies with BRAF^V600^ and MEK inhibitors [25]. Several drugs have been accepted by the FDA (Food and Drug Administration) and EMA (European Medicines Agency) for the treatment of melanoma patients in unresectable stages of tumor development [25,26,27,28,29]. Although targeted therapies are very promising, they are challenged by intrinsic resistance and the development of acquired resistance in approximately one-half of the melanoma patients within a few months [30,31,32,33,34,35,36,37].

Depending on the tumor type and its major driving oncogenes, the FGF/FGFR signaling can be differently utilized by tumor cells to maintain their malignancy and can be variably affected by therapeutics, especially those targeting tumor-specific oncoproteins [16,38]. This review article summarizes the current knowledge on the fibroblast growth factor receptor signaling in skin cancers with a focus on melanoma.

## 2. Fibroblast Growth Factor Receptor Signaling Pathway in Normal Skin

The skin is a complex organ. The epidermal layer is composed of keratinocytes, melanocytes, and inflammatory cells. The dermal layer is made up of fibroblasts, hair follicles, blood vessels, sweat glands, nerve endings, monocytes/macrophages, and T cells. Melanocytes are neural crest-derived cells, and mature melanocytes are anchored to the basement membrane, which holds together epidermis and dermis. Under physiological conditions, receptor signaling pathways are tightly controlled to provide skin homeostasis. Melanocytes produce pigment-containing melanosomes and transport them through dendrites to keratinocytes [39]. Melanocytes are under control of keratinocytes, and each melanocyte is connected with more than 30 keratinocytes to form the melanin unit protecting the skin from UV light. Several secreted factors, such as FGF2 (also known as bFGF, basic fibroblast growth factor), stem cell factor (SCF), hepatocyte growth factor (HGF), melanocyte stimulating hormone (MESH), and endothelin (ET), have been detected in the normal skin [40]. When secreted in response to ultraviolet (UV) light, they contribute to the stimulation of pigmentation and melanocyte proliferation via their receptors on melanocytes, e.g., FGF2 is secreted by UVB-exposed keratinocytes [41]. In general, melanocytes rarely undergo mitosis. Melanocytes mainly proliferate during the expansion of the skin surface in childhood or wound healing, and at a low rate upon stimulation by sunlight exposure. To undergo mitosis, they detach from keratinocyte and the basement membrane, and their dendrites are drawn out. After division, they migrate along the basement membrane to form a new melanin unit with keratinocytes. In normal skin, FGF2 is secreted by fibroblasts in the dermal layer and keratinocytes in the epidermis. FGF2 is highly mitogenic for melanocytes in vitro [41,42,43]. FGF2 could induce a transformed phenotype in melanocytes [44]; however, in another study, overexpression of FGF2 in skin xenografts induced hyperpigmentation and proliferation of melanocytes but no malignant transformation [45]. Melanoma-like lesions appeared when FGF2 overexpression was combined with UVB but not UVA [45,46]. FGF2 was also shown to be a melanocyte survival factor [47]. It was demonstrated that dermal nevus cells were able to survive in 3D type 1 collagen culture, whereas normal melanocytes underwent apoptosis unless the collagen culture was supplemented with FGF2 [47]. It was suggested that the higher level of FGF2 in the microenvironment of dermal nevus-derived melanocytes allowed melanocytes to adapt to grow in the dermis, which might be important for the development of melanoma.

## 3. Fibroblast Growth Factor Receptor Signaling in Melanoma and Other Skin Cancers

The development and progression of melanoma is a complex process that usually consists of six steps: 1. common acquired melanocytic nevi; 2. melanocytic nevi with hyperplasia; 3. melanocytic nevi with dysplasia; 4. the radial growth phase (RGP) of primary melanoma; 5. the vertical growth phase (VGP) of primary melanoma; 6. metastatic melanoma [48]. In addition to being produced by neighboring fibroblasts and keratinocytes, FGF2 can be synthesized by cells in nevi and melanoma cells. In dysplastic nevi, moderate to high FGF2 levels were detected, whereas in common acquired nevi, contradicting data were obtained [49,50]. Melanoma progression was shown to be accompanied by increased FGF2 expression [51]. It was demonstrated that the switch to growth factor independence could distinguish RGP lesions from tumorigenic VGP primary melanoma [52]. A comparison of FGFR expression revealed an absent or very weak expression in nevi and diverse expression levels in primary and metastatic melanomas [53]. In a more recent study, FGF2 was detected in 72% of non-dysplastic nevi and only in 18% of dysplastic nevi [54]. Co-expression of FGF2 and FGFR1 was found in 60% of non-dysplastic nevi and in 18% of dysplastic ones. FGFR1 was detected in 86%, whereas FGF2 in 45% of primary melanomas [54]. In 2/3 of melanomas, FGF2 was also expressed by keratinocytes in the epidermis, and FGFR1 was commonly detected in the epidermis [54]. As suggested by the authors of the latter study, the discrepancies between the results obtained by different laboratories may be due to different primary antibodies used to detect FGF2 and FGFR1.

It has been demonstrated that melanoma cells stimulate their FGFRs in an autocrine manner [49,50,55,56,57,58,59,60]. An in vitro study showing melanoma cell independence on exogenously added growth factors suggested that the growth factors received by melanoma cells in a paracrine manner play a minor role [61]. Patient-derived BRAF^V600E^ melanoma cells could grow without serum and exogenous growth factors, FGF2, EGF (epidermal growth factor), and HGF (hepatocyte growth factor), for at least 4 months without substantial changes in viability and cell phenotype. Neither the cell cycle nor the activity of pathways important for melanoma maintenance, such as MAPK-ERK, WNT (wingless/integrated)/β-catenin, and NF-κB (nuclear factor kappa B), were affected by the absence or presence of FGF2, HGF, and EGF used alone or in combination. More interestingly, lack of exogenous growth factors did not influence acute cell response to vemurafenib and trametinib, targeting BRAF^V600E^ and MEK1/2, respectively [61]. Although the expression of growth factors in melanoma cells was not blocked in this study and endogenous and exogenous level was only measured for HGF, another growth factor considered as important for melanoma [62], it is possible to conclude that endogenous growth factors released by melanoma cells and/or mutation-driven activation of the MAPK-ERK signaling pathway seem to be sufficient to maintain the major functions in a subset of melanoma cell lines. Mutation status might be also important for the sensitivity of melanoma cells to growth factors. It was demonstrated that wild-type melanoma cell lines were more responsive to FGF2 than BRAF mutant or NRAS mutant cell lines [63].

Co-expression of the pair FGF2 and FGFR1 in melanoma was extensively characterized in the 1980s and 1990s [64,65]. Early studies indicated that FGF2/FGFR1 might be of importance for autocrine growth control and melanoma progression [42,44,53,66,67]. Suppression of the FGFR1 activity inhibited cell proliferation and survival and induced cell differentiation [68,69]. Down-regulation of *FGF2* in melanoma cells inhibited proliferation and colony formation [42,66]. Melanoma cells could not survive in vitro and in vivo if FGFR1 or FG2F were targeted [65,66,70,71,72]. Inhibition of FGF2 and FGFR1 in melanoma cells in vivo induced apoptosis and blocked intratumoral angiogenesis and tumor growth [65,71]. More recent studies have shown that the majority of melanoma cell lines concomitantly overexpressed FGF2, FGF5, and FGF18 and diverse isoforms of FGFRs [73]. For instance, FGF2 transcript levels were more than 100-fold higher in half of melanoma cell lines than in normal melanocytes [73], and FGF5 in 1/3 of melanoma cell lines [74]. FGF5 protein has been recently shown to be endogenously overexpressed in a subset of melanoma cell lines and in more than 60% of benign nevi and melanoma specimens [74]. These results were supported by data from the Cancer Genome Atlas [74]. Blockade of FGF/FGFR signaling by genetic constructs or kinase inhibitors inhibited melanoma growth, and synergistic anti-melanoma effects were obtained in vitro and in vivo if BRAF inhibitors were combined with FGFR inhibition [73].

Another interesting study suggesting that inhibition of the FGF/FGFR signaling might improve the response to targeted therapies has been published most recently [75]. In metastatic uveal melanoma (UM) that developed resistance to inhibitors of bromodomain and extra-terminal domain (BET) proteins, elevated levels of stromal FGF2, but not other factors, were detected. In addition, BET inhibitors could enhance FGFR expression in UM cell lines and patient tumor samples, while FGFR inhibitors (AZD4547 and BLU9931) reversed the effects of stromal FGF2. These results strongly suggest that co-inhibition of the FGF2/FGFR signaling pathway is necessary to prevent the development of resistance and improve the efficacy of BET inhibitors [75]. It was shown in a mouse model that FGF2 was secreted by liver cells at the site of metastasis. This may indicate that paracrine secretion of growth factors by organ-specific cells in the tumor microenvironment and elevated expression of FGFRs in melanoma cells are resistance mechanisms reducing treatment efficacy.

It has been shown that the FGF2/FGFR1 signaling is also crucial for melanoma angiogenesis as FGF2 secretion by melanoma cells could induce the mitogenic effects on endothelial cells and fibroblasts [65]. In VGP primary melanomas, co-expression of FGF2 and FGFR1 is significantly associated with increased density of microvessels [76]. FGF2 is known to stimulate the expression of vascular endothelial growth factor (VEGF) in vascular endothelial cells [77]. In a mouse model, when FGF2 was injected at the melanoma inoculation site during the initial phase of tumor growth, both, VEGF-A-dependent neovascularization of the host stroma and melanoma metastasis were enhanced [78]. Cancer cell metabolism substantially contributes to angiogenesis, metastasis, and suppression of the immune system. One of the important factors is lactate, which is secreted by cancer cells to the tumor microenvironment (TME), and it is taken up by endothelial cells. This, in turn, results in up-regulation of FGF2 and vascular endothelial growth factor receptor 2 (VEGFR2) signaling and induction of angiogenesis [79].

Besides melanocyte-originated melanoma, skin cancers include keratinocyte-originated seborrheic keratosis (SK), squamous cell carcinoma (SCC), and basal cell carcinoma (BCC), among others. Abnormal FGF/FGFR3 signaling has been observed in SK [80,81,82,83,84]. FGFR3 activation is, however, insufficient to drive skin tumors, such as SCC, and additional genetic and/or microenvironmental factors are required for epidermis to progress malignancy [85]. Autocrine FGF10/FGFR2 signaling may promote cutaneous SCC, representing about 25% of non-melanoma skin cancers [86]. It was shown in a mouse model of SCC that the enhanced mTOR (a mammalian target of rapamycin) signaling obtained in keratinocytes by deletion of *Pten* (phosphatase and tensin homolog deleted on chromosome 10) strongly enhanced the level of Fgf10 protein, and *Pten* deletion-induced skin cancers were inhibited by epidermal *Fgfr2* deletion [86]. Moreover, in clinical samples, almost all SCC specimens showed a PTEN loss and an increase in FGF10 when compared to normal skin from a patient undergoing abdominoplasty. A previously published report suggested a tumor-suppressive function of the FGFR2 signaling in the skin [87]. These two opposing functions of FGF10 signaling—tumor suppressive and oncogenic—might be explained by different magnitudes of signaling [86]. In addition, the oncogenic function of FGF10/FGFR2 signaling might be especially potent in PTEN-deficient epidermis. It has been shown that the activity of PI3K in PTEN-deficient skin lesions is enhanced by the FGF-activated RAS-MAPK signaling [88].

It has been reported that FGF2 is overexpressed in another nonmelanocytic skin cancer, BCC [89]. FGF2 could induce angiogenesis and survival in BCC via STAT3 and PI3K/AKT pathways [90]. Dobesilate, the inhibitor of FGF, reduced the level of phosphorylated STAT3 in BCC, which was accompanied by the promotion of apoptosis, and inhibition of proliferation and angiogenesis [91]. In addition, dobesilate showed high efficacy in the topical treatment of two BCC patients [91].

## 4. Mutations of Genes Encoding FGFRs in Melanoma and Other Skin Cancers

The primary mechanism for abnormal signaling is connected with point mutations and chromosomal translocations that often result in constitutive dimerization and kinase activation of growth factor receptors, including FGFRs [92,93]. FGFR overexpression can be also achieved by repression of miRNAs targeting FGFR transcripts or up-regulation of long-non-coding RNAs (lncRNAs) sequestering FGFR-targeting miRNAs [94]. Elements of FGF/FGFR signaling are very frequently mutated kinases in cancers [18,95], and DNA sequencing of cancer specimens has revealed a plethora of mutations in genes encoding FGFRs and FGFs.

According to an analysis based on data from cbioportal at http://www. cbioportal.org/public-portal (accessed November 2014) by Helsten et al. [17], the frequency of aberrations in *FGFR* reached 20% in melanoma, and it is lower than in bladder urothelial carcinoma (35%) or lung, squamous cell carcinoma (about 27%). The frequency of *FGF* alterations in melanoma is about 38%, and the highest frequency is observed in head and neck squamous cell carcinoma (nearly 54%). Based on a large-scale high-throughput study, the same group showed that melanoma exerts one of the lowest frequencies of *FGFR* aberrations among all tested cancers [18].

Amplification of genes encoding FGFR can be found in many cancers, including head and neck squamous cell carcinoma, non-small-cell lung, breast and gastric cancers [96,97,98,99,100], but not in melanoma.

Chromosomal translocations can lead to fusion proteins with oncogenic potential. The most common fusion partner for *FGFR*, especially *FGFR2* and *FGFR3*, is *TACC3* encoding transforming acidic coiled-coil containing protein 3 [18]. This fusion was originally identified in glioblastoma [101] and was recently shown to cluster within transcriptional subgroups that have metabolic functions [102]. *FGFR3-TACC3* fusion leads to a loss of the miR-99a binding site, which results in an up-regulation of fusion protein translation [103]. The first case of melanoma harboring an *FGFR3-TACC3* fusion has been reported recently [104].

Non-synonymous somatic point mutations occur most commonly in *FGFR3*. Several somatic activating mutations have been found in *FGFR3* in seborrheic keratoses, one of the most frequent benign epidermal tumors in older patients [81,105]. It has been demonstrated that one of these mutations in the extracellular domain of FGFR3, leading to the Ser249Cys substitution, can induce benign skin tumors in mouse epidermis [81]. The mutation leading to Arg248Cys has been also detected in adenoid seborrheic keratoses, very common benign skin tumors [106]. Mutations localized in the positions 248 and 249, with cysteine substitution, are thought to increase the stability of the FGFR3 dimer, which stimulates the receptor in a ligand-independent manner [107]. Stimulated receptor induces the transcription factor forkhead box N1 (FOXN1), which triggers the expression of FGFR3, and this feedback loop may antagonize RAS activity by promoting differentiation [84]. More recently, it has been reported that activating mutations in *FGFR3* leading to Arg248Cys, Ser249Cys, and Gly697Cys can cause mild hyperplasia in the skin but are insufficient to induce benign or malignant skin cancers, including SK and SCC [85]. The Lys650Met FGFR3 mutation has been detected in acanthosis nigricans (AC), a benign skin tumor [108,109]. A mutation leading to the Gly380Arg substitution has been found in *FGFR3* of cutaneous SCC [110]. The Gly380Arg FGFR3 mutation increases the ligand-independent phosphorylation of FGFR3, which causes constitutive activation of the downstream signaling pathways [111]. Interestingly, activating *FGFR3* mutations, mainly Arg248Cys, can be also found in epidermal nevi, a common congenital skin lesion with an incidence of 1 in 1000 people [112]. It has been suggested that a large part of epidermal nevi results from mosaicism of activating *FGFR3* mutations in the epidermis or postzygotic mutations in early embryonic development [112].

Pro252Arg/Ser/Thr somatic mutations in the fragment *FGFR1* encoding its extracellular domain were identified among genetic drivers in human melanoma cell lines [113]. These gain of function mutations might increase receptor-ligand binding affinity by reducing the dissociation rate of the FGF ligand [114,115].

Similarly, as for genotypes of FGFR4 in breast, colorectal, and lung cancers [116,117], an SNP (rs351855) that results in Gly388Arg substitution can be found in melanomas. Fifty-five percent of melanoma patients had a homozygous Gly388 allele, while 45% harbored at least one Arg388 allele [118]. Most recently, this mutation was found in three out of eight patient-derived melanoma cell lines [119]. Arg388 allele correlated with tumor thickness and was earlier associated with increased cell motility and invasiveness [116]. It has been shown that the Gly388Arg substitution induces conformational changes in receptor structure, which results in the enhanced association of FGFR4 with STAT3 [120]. The analysis of the literature has shown the lack of unambiguous evidence of the pro-tumorigenic and pro-metastatic impact of FGFR4 Arg388 allele in the majority of cancers, including melanoma [121].

Although loss-of-function mutations are not common in cancer, these type of aberrations (more than 20 different point mutations) have been found in *FGFR2* in some melanoma cell lines and uncultured primary and metastatic tumors [122]. Therefore, FGFR2 was classified as melanoma suppressor [123]. Loss of receptor activity might be achieved through diverse mechanisms, such as loss of ligand binding affinity, retention in the endoplasmic reticulum, impaired receptor dimerization, and loss of tyrosinase activity [122]. It has been suggested that the inhibition of FGFR2 activity contributes to melanoma; however, the mechanism(s) is not clear, and reintroduction of *FGFR2* failed to suppress melanoma cell proliferation [122]. As FGFR2 could induce melanoma growth arrest through interaction with stroma, its inhibition might promote melanoma invasion [123].

FGFR mutations reported in skin cancer patients and cell lines, and their relative location on the proteins are summarized in Table 1.

## 5. FGF/FGFR Signaling in Resistance to Targeted Therapies

The mechanisms of intrinsic and acquired resistance of melanoma to targeted therapies are diverse [36,124,125,126,127,128]. Reduced drug sensitivity of melanomas has been associated with increased expression/activities of multi-RTKs, including epidermal growth factor receptor (EGFR), cellular mesenchymal-epithelial transition factor (c-MET, hepatocyte growth factor receptor), platelet-derived growth factor receptor beta (PDGFRB), insulin growth factor 1 receptor (IGF1R), and AXL receptor tyrosinase kinase (AXL) [127,129,130,131,132,133,134,135,136,137,138,139,140]. FGF was among growth factors that were capable of attenuating the antiproliferative effects of vemurafenib, which was accompanied by the reduced ability of vemurafenib to inhibit pMEK [140]. This effect was reversed by FGFR inhibitor PD173074 [140]. FGF1 reactivated pERK in most melanoma cell lines treated with BRAF^V600E^ inhibitor in another study [132].

**Figure 2:**
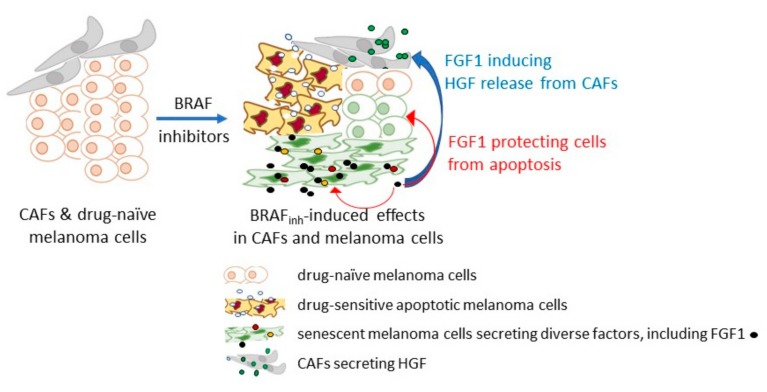
A simplified schematic illustration of fibroblast growth factor 1 (FGF1)-induced resistance to BRAF (v-Raf murine sarcoma viral oncogene homolog B) inhibitors in melanoma cells. BRAF inhibitors, e.g., vemurafenib and mitogen-activated protein kinase (MEK) inhibitors, e.g., trametinib can induce diverse changes in drug-naïve melanoma cells, including apoptosis and premature senescence, while some melanoma cells remain unaffected. Thus, targeted therapy enhances the phenotypic heterogeneity of the neoplastic lesion in the tumor niche. Melanoma cells exerting the senescence-associated secretory phenotype (SASP) secrete several cytokines and growth factors, including FGF1. FGF1 released by senescent melanoma cells can stimulate melanoma cells and neighboring cells. Melanoma cells become protected from apoptosis, whereas cancer-associated fibroblasts (CAFs) are stimulated to secrete hepatocyte growth factor (HGF). Although these mechanisms are not universal and are not observed in all tested melanoma cell lines, they can contribute to drug resistance in a subset of melanomas. The scheme was prepared based on the study of Grimm et al. [143].

In drug-naïve BRAF^V600^ melanoma cells, the RTK expression is low, probably because the strong endogenous BRAF/NRAS mutation-driven activation of the MAPK-ERK signaling pathway selects, against cells with active RTK signaling, to prevent senescence [125]. FGFR1 and to some extent FGFR4 are exceptional in contrast to FGFR2-3, and these receptors are expressed in the majority of melanomas [54,73,141]. *FGFR1* has been listed among resistance-associated genes expressed transiently in melanoma cells [142]. As suggested by Grimm et al., elevated FGFR1 expression allows melanoma cells to react immediately on ligands that are induced by drugs [143]. A therapy-induced senescence-like state is one of the options for melanoma cells to survive the treatment (Figure 2).

It has been demonstrated that the inhibition of BRAF^V600^ can induce a secretome with stimulating effects on both adjacent drug naïve melanoma cells and fibroblasts [143]. FGFs were found among senescence-associated factors expressed and released by melanoma cells in response to BRAF and MEK inhibition. It has been shown that FGF1 could limit the pro-apoptotic activities of drugs and activate fibroblasts to secrete HGF [143]. This contributes to “minimal residual disease”, while continued BRAF^V600^ inhibition may induce secondary resistance mechanisms [143]. When inhibitors of FGFR have been applied in parallel to BRAF inhibitors, the resistance of melanoma cells was diminished. As the regulation of FGF1 was jointly mediated by FRA1 repression and activation of the PI3K/AKT signaling pathway, which is considered as the compensatory pathway activated by targeted therapy against BRAF^V600/^MEK [144,145], obtained results suggest that targeting FGF/FGFR signaling pathway might be considered as the opportunity to block the compensatory pathway.

Considering cross-talk between the FGF/FGFR signaling and other oncogenic signaling pathways, the role of FGF/FGFR signaling in the development of acquired resistance to therapies targeting oncoproteins is conceivable. FGFR3 activation has been proposed as a mechanism of acquired resistance to vemurafenib in BRAF^V600E^ melanomas [146] and to cetuximab in KRAS wild type SCC [147]. Inhibition of the FGFR3/RAS axis could restore the sensitivity of resistant melanoma cells to vemurafenib [146].

A new variant of *FGFR2* with a mutation leading to Gly542Glu was reported in BRAF^V600E^ melanoma patient during ongoing treatment with dabrafenib, a BRAF^V600E^ inhibitor, as an example of intratumor clonal evolution resulting in the development of resistance [148].

## 6. FGF and FGFR as Molecular Prognostic Markers

Several factors, such as dose of sun exposure, age, gender, fair skin phenotype, and previous keratinocyte skin cancers, are considered as causal factors of initiation and progression of melanoma, with UV-induced damage of DNA as the primary cause of melanoma development [149,150,151]. However, the incidence of melanoma in childhood and at sites that are minimally exposed to the sun are also registered [152,153]. There is hope that genome-wide studies will help to identify the heritable and environmentally-triggered contributors to melanoma risk [153,154,155,156,157]. Another important issue is to correctly prognose the melanoma progression. The 10-year survival among patients diagnosed with melanoma localized to the skin (stage I-II) is high if surgical treatment is applied. But still, it is unclear how to predict the recurrence in melanoma patients. Therefore, the development of prognostic tools applied at the time of diagnosis and surgery, which could select stage I-II melanoma patients of high recurrence risk for adjuvant therapy, might further reduce the burden of untreated melanomas. As melanoma is a highly heterogeneous tumor [32], currently used clinicopathologic prognostic markers do not account for all registered variability connected with melanoma progression. Also, none of the key mutations that emerged as the result of genome-wide genetic analysis could be unambiguously linked to melanoma progression. Therefore, molecular classifiers predictive of melanoma outcomes are in focus of several studies, and several models, including MelaPRO and GenoMELPREDICT, have been developed, and genetics consortium GenoMEL (www.genomel.org) has been established.

Loss-of-function mutations in *FGFR2* and activating mutations in *FGFR4* have been connected with melanoma. In a large nested case-control study of Caucasian women, however, neither mutations in *FGFR2* nor in *FGFR4* has been found as contributing to an inherited predisposition to three skin cancer types, including melanoma, SCC, and BCC [158].

Therefore, it has been suggested that rather than serving as predisposition indicators, genetic aberrations in *FGFR2* and *FGFR4* might be used as potential biomarkers of melanoma progression. FGFR4 has been detected at the protein level in 45% of 137 melanoma specimens [118]. It has been demonstrated that its expression correlated with pTNM melanoma stage (*p* = 0.046), vascularity (*p* = 0.001), microulceration (*p* = 0.009), metastases (*p* = 0.025), overall survival (*p* = 0.047), and disease-free survival (*p* = 0.046). Moreover, Arg388 polymorphism was present in 45% of melanoma patients. The FGFR4Arg388 allele has been associated with intensified melanoma motility and invasiveness [116]. Therefore, both, expression of *FGFR4* and Arg388 genotype was suggested as potential biomarkers for the progression of melanoma, although no further study has been published to extend these findings.

Analysis of 418 manuscripts revealed several proteins that could potentially stratify melanoma, and FGFR1 was listed among 67 proteins categorized as contributing to self-sufficiency in growth signals [159]; although in an earlier study, there was no correlation between expression of FGFR1 and the usual clinicopathological features of melanoma [141].

Up to date, none of the elements of the FGF/FGFR signaling pathway is included as the biomarker in the diagnosis of melanoma.

## 7. Targeting Aberrant FGF/FGFR Signaling in the Clinic

The FGFR inhibitors are still under development, and there are currently no FGFR-targeted therapies, which are approved for the treatment of cancer patients [160,161,162]. Results of early-phase clinical trials from different cancers suggest that selective FGFR inhibitors could be useful in treating patients with FGFR fusions and selected patients with *FGFR2* amplification [160]. It is thought that inhibitors of FGFRs evoke effects on cancer cells but also indirectly through paracrine signaling on angiogenesis and immune evasion [94]. 

While in the preclinical melanoma models, a small molecule FGFR inhibitor SU5402 has demonstrated activity [73], it was not tested in clinical trials. In a phase I study, lenvatinib, an oral tyrosine kinase inhibitor, was evaluated in melanoma patients, where tumor shrinkage was observed, but many adverse effects were also reported [163].

In the ongoing LOGIC-2 phase II clinical trial (https://clinicaltrials.gov/ct2/show/NCT02159066) for advanced BRAF melanomas, one of the FGFR1–3 selective inhibitors, BGJ398 (Infigratinib), was included as the third agent used in combination with LGX818 (Encorafenib), a potent BRAF inhibitor, and MEK162 (Binimetinib), a selective inhibitor of MEK. The study design allows applying BGJ398 after progression from therapy against BRAF/MEK. The estimated LOGIC-2 study completion date for advanced melanoma patients is 2022. BGJ398 was/is investigated in several clinical trials (phase I and II), and, so far, the analysis of the results indicated a substantial variability in response rates. In a phase I study, it demonstrated partial response in four patients with *FGFR1*-amplified non-small-cell carcinoma (NSCLC) and stable disease in 14 patients [164]. Only one breast cancer patient with *FGFR1*-amplified showed tumor regression, and partial responses were reported in *FGFR3*-mutated bladder cancer, while in one patient with hepatocellular carcinoma (HCC) and in one patient with cholangiocarcinoma (CCA), both with *FGFR2-BICC1* (bicaudal C homolog 1) fusion gene, tumor shrinkage was observed [155]. In the ongoing phase II clinical studies, BGJ398 is investigated in advanced-stage gastrointestinal stromal tumors (https://clinicaltrials.gov/ct2/show/NCT02257541) and advanced CCA (https://clinicaltrials.gov/ct2/show/NCT02150967) and other hematological and solid cancers with FGFR genetic alterations (https://clinicaltrials.gov/ct2/show/NCT02160041). Three patients with CCA, who progressed on BGJ398, acquired recurrent polyclonal point mutations in the kinase domain of fusion FGFR2, which led to the development of drug resistance [165].

Several challenges are recognized in clinical trials applying FGFR inhibitors, including dosing limitations, diversity of the mechanisms activating FGF/FGFR signaling, and difficulties in prospective selection of patients with specific aberrations in FGFRs.

## 8. Conclusions

The FGF/FGFR signaling does not markedly contribute to the development of melanoma as it is suppressed by the overactivated RAS/BRAF/MEK/ERK (MAPK) signaling pathway. When BRAF/MEK inhibitors are applied, and the pathway is blocked, the FGF/FGFR signaling might be considered as one of the mechanisms that lead to the development of resistance limiting the success of targeted therapies against BRAF and MEK. First attempts are undertaken in the clinics (LOGIC-2) to find out whether targeting the FGF/FGFR signaling pathway with BGJ398 (Infigratinib) may be a good therapeutic strategy in combination with BRAF/MEK inhibitors.

## Figures and Tables

**Table 1:** Mutations of genes encoding fibroblast growth factor receptors (FGFRs) in melanoma and other skin cancers.

Cancer	Gene	Encoded Substitution/Fusion	Refs
acanthosis nigricans	*FGFR3*	K650M	[108,109]
adenoid seborrheic keratoses	*FGFR3*	R248C	[106]
squamous cell carcinoma	*FGFR3*	G380R	[110]
melanoma	*FGFR1*	P252R/S/T	[113]
	*FGFR2*	S24F, V77M, E160A, H213Y, E219K, G227E, V248D, R251Q, G271E, G305R, T370R, W474X, E475K, D530N, E574K, E636K, M640I, I642V, A648T, S688F, G702S, P708S, R759X, R759Q, L770V	[122]
	*FGFR3*	*FGFR3-TACC3* fusion	[104]
	*FGFR3*	P451S	[119]
	*FGFR4*	G388R	[118,119]
